# Dissecting the recruitment and self-organization of αSMA-positive fibroblasts in the foreign body response

**DOI:** 10.1126/sciadv.add0014

**Published:** 2022-12-21

**Authors:** Maria Parlani, Matthew L. Bedell, Antonios G. Mikos, Peter Friedl, Eleonora Dondossola

**Affiliations:** ^1^ Department of Genitourinary Medical Oncology and David H. Koch Center for Applied Research of Genitourinary Cancers, The University of Texas MD Anderson Cancer Center, Houston, TX 77030, USA.; ^2^ Radboud University Medical Center, Nijmegen, Netherlands.; ^3^ Department of Bioengineering, Rice University, Houston, TX 77030, USA.; ^4^ Cancer Genomics Centre (CGC.nl), 3584 Utrecht, Netherlands.

## Abstract

The foreign body response (FBR) is a clinically relevant issue that can cause malfunction of implanted medical devices by fibrotic encapsulation. Whereas inflammatory aspects of the FBR have been established, underlying fibroblast-dependent mechanisms remain unclear. We here combine multiphoton microscopy with ad hoc reporter mice expressing α–smooth muscle actin (αSMA) protein to determine the locoregional fibroblast dynamics, activation, and fibrotic encapsulation of polymeric materials. Fibroblasts invaded as individual cells and established a multicellular network, which transited to a two-compartment fibrotic response displaying an αSMA cold external capsule and a long-lasting, inner αSMA hot environment. The recruitment of fibroblasts and extent of fibrosis were only incompletely inhibited after depletion of macrophages, implicating coexistence of macrophage-dependent and macrophage-independent mediators. Furthermore, neither altering material type or porosity modulated αSMA^+^ cell recruitment and distribution. This identifies fibroblast activation and network formation toward a two-compartment FBR as a conserved, self-organizing process partially independent of macrophages.

## Teaser

Fibroblast recruitment in the foreign body response is a conserved, self-organizing process partially independent of macrophages.

## INTRODUCTION

The foreign body response (FBR) is the end-stage result of inflammatory and wound healing processes to shield external material after intrusion (*1*). This pathophysiological process has obtained increasing clinical attention, as it leads to inflammation and fibrotic encapsulation of medical implants in patients, compromising both long-term tissue integration and function (*1*, *2*). The interplay between inflammatory cells, vascular networks, and activated fibroblasts is critical to mount a response. The FBR starts with vascular damage and plasma protein engagement on the implant surface (*3*). This is followed by neutrophilic inflammation and the recruitment of monocytes, which become activated to macrophages and, by fusion, foreign body giant cells (FBGCs) (*4*). In parallel, a neoangiogenic vascular network is formed, and fibroblasts are activated to deposit a collagenous capsule (*1*, *5*). Although the role of the immune compartment has been thoroughly dissected, including both the cellular and molecular nature of the infiltrating cells (*4*, *6*–*11*), the principles governing the engagement of fibroblasts during the FBR in vivo have not been systematically investigated (*12*, *13*).

Interstitial fibroblasts represent central effectors involved in tissue homeostasis, remodeling, and wound healing processes (*14*, *15*) and present distinct molecular and morphological characteristics according to their organ of origin, body site, and spatial location (*16*). Fibroblast populations further comprise blood vessel–associated pericytes (*17*) and myofibroblasts, which are activated fibroblasts that critically contribute to tissue repair (*18*, *19*). Upon tissue damage, quiescent fibroblasts are engaged by transforming growth factor–β (TGFβ) and platelet-derived growth factor (PDGF) produced by inflammatory cells (*20*, *21*). When activated, myofibroblasts reversibly up-regulate α–smooth muscle actin (αSMA), which confers contractile properties and modifies their biosynthetic, secretory, and migratory functions (*14*, *19*, *22*). Besides depositing collagens and fibronectin, myofibroblasts produce metalloproteinases that remodel the extracellular matrix and release growth factors and proinflammatory cytokines including TGFβ, PDGF, vascular endothelial growth factor α, hepatocyte growth factor, and tumor necrosis factor, which foster the fibrotic process (*14*, *19*).

At the end of a transient fibrotic process, such as wound healing, myofibroblasts become eliminated by apoptosis (*19*). If the insult persists, however, the repair response chronicizes and causes long-lasting fibrosis (*23*). In this case, fibroblasts may acquire further properties such as permanent hyperactivation, increased proliferation, and functional diversification, including specialized matrix remodeling ability, a highly secretory phenotype, and increased immunomodulatory functions (*14*, *16*, *24*). Now, our understanding of the critical contribution of fibroblasts to the FBR is still unclear. Specifically, the kinetic steps of fibroblast recruitment, the timing and location of activation, and their self-organization to achieve fibrotic encapsulation of the foreign material have not been observed in vivo, mainly because of the inability to longitudinally track this dynamic process with cellular resolution in situ in living organisms (*12*, *15*, *25*).

Experimental approaches to probe the FBR in small animals are mostly based on ex vivo endpoint analysis, which reveals the cellular content and extent of fibrosis from two-dimensional (2D) sections and the activity of the wound using macroscopic imaging (*1*). Although informative, these strategies lack sensitivity, time resolution, and insights into 3D organization and evolution of this process. Intravital microscopy, including nonlinear intravital multiphoton microscopy (iMPM), complements these approaches by providing mechanistic, 3D, and time-resolved insight into the position, dynamics, and function of single cells at subcellular resolution and in real time (*8*, *9*, *25*–*27*). In combination with window models in fluorescent reporter mice, iMPM delivers real-time access to the microenvironmental niches that mediate the FBR progression, including immune and stromal cell recruitment, matrix remodeling, and neovessel anatomy and function (*25*, *26*, *28*). Using iMPM, we have recently developed noninvasive imaging procedures for monitoring the development of the FBR in intact green fluorescent protein (GFP) reporter mice and identified that immune infiltrating cells dynamically engage with implanted materials over time, followed by collagen deposition and blood vessel formation (*26*). In this work, we extended iMPM to multiparameter fluorescent reporter mouse models and investigated the step-wise recruitment, activation, and self-organization of fibroblast networks in the FBR. We further addressed the impact of macrophages and material composition on the temporal progression of fibroblast activation and function and fibrosis outcome.

## RESULTS

### Fibroblast activation upon material implantation

**Fig. 1.**
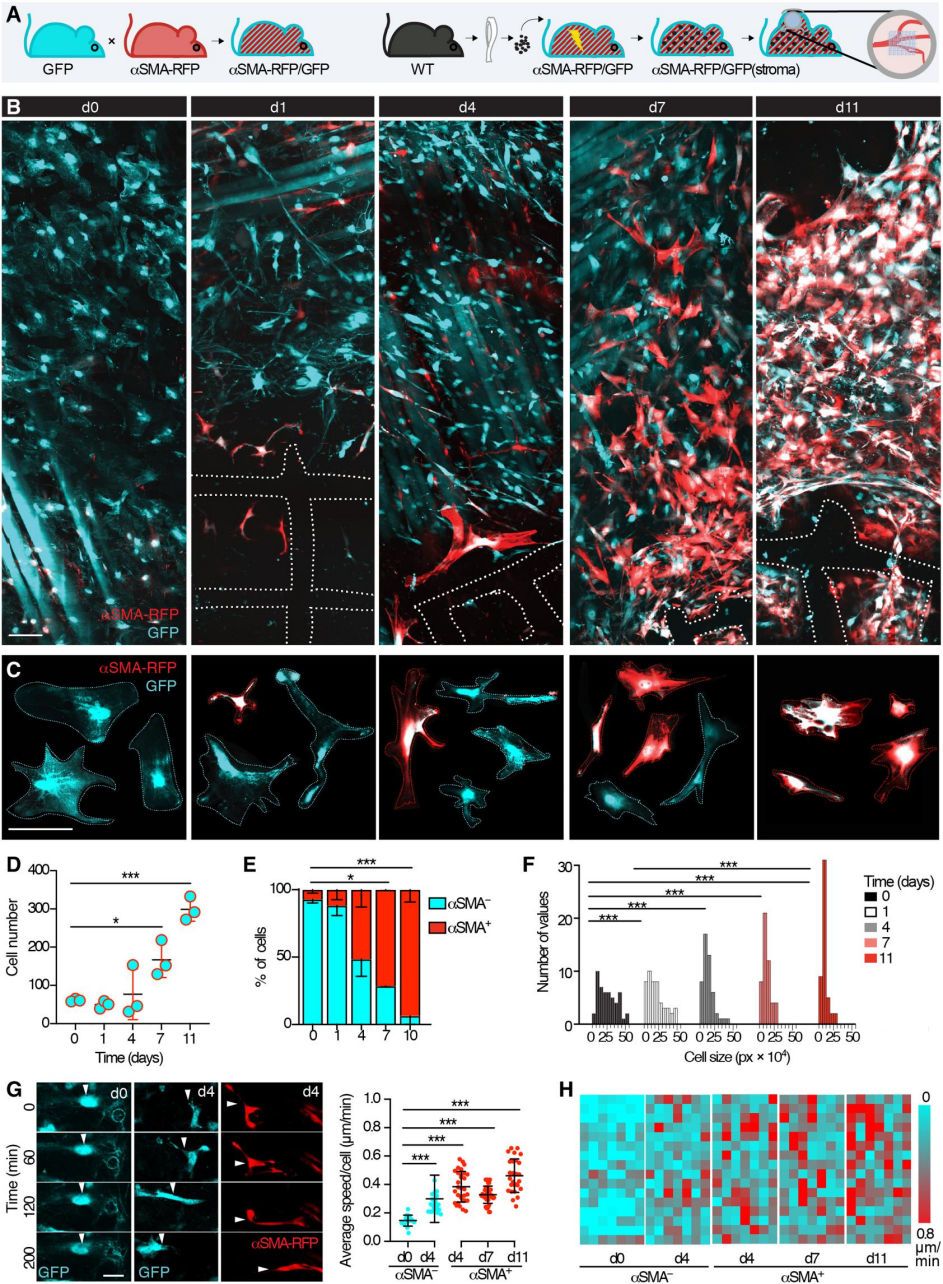
Monitoring fibroblast activation and dynamics upon material implantation by longitudinal iMPM. (**A**) Schematic representation of the model. An αSMA-RFP/GFP model is generated by breeding followed by bone marrow transplant, resulting in the αSMA-RFP/GFP(stroma) reporter mouse, which is implanted with a dorsal skinfold chamber (DSFC) and a PCL scaffold. WT, wild type. (**B**) Longitudinal intravital imaging of the fibroblast recruitment on the day of the scaffold implantation and after 1, 4, 7, and 11 days. Dotted lines, scaffold position; GFP^+^ cells, cyan; αSMA-RFP^+^ cells, red. Scale bar, 50 μm. (**C**) Morphology of representative quiescent and activated fibroblast over time (days 0, 1, 4, 7, and 11 after the scaffold implantation). GFP^+^ cells, cyan; αSMA-RFP^+^ cells, red. Scale bar, 50 μm. (**D**) Average number of fibroblasts counted over time (360 μm by 360 μm by 50 μm, three mice per time point). (**E**) Percentage of αSMA^−^ (cyan) and αSMA^+^ (red) fibroblasts over time (360 μm by 360 μm by 50 μm, three mice per time point). (**F**) Cell size over time with frequency distribution over time; *n* = 3 mice, 50 cells per time point. (**G**) Sequential frames obtained at different time points of representative αSMA^−^ cell before scaffold implantation, an αSMA^−^ cell and an αSMA^+^ cell 4 days after the scaffold implantation. The dynamics of αSMA^−^ and αSMA^+^ cells at different time points were monitored by time-lapse intravital microscopy and analyzed by single-cell tracking. Cell speed over time is shown, *n* = 25 to 40 cells in three mice per time point. Scale bar, 10 µm. (**H**) Heatmaps of the speed from seven representative cells per time points are shown. *P < 0.05, **P < 0.01, and ***P < 0.001; one-way analysis of variance (ANOVA) followed by Tukey’s honestly significantly different (HSD) post hoc test.

To monitor fibroblast engagement and fate during the FBR in the deep dermis in vivo, we applied an established intravital window model combined with fibrous polycaprolactone (PCL) scaffold implantation (*26*) in dual-color reporter mice. C57BL/6 ubiquitin (UBC)-GFP mice expressing GFP ubiquitously were crossed with C57BL/6 [Acta2–red fluorescent protein (RFP)]1Rkl/J mice expressing RFP in all cells that produce αSMA to generate an αSMA-RFP/GFP model (Fig. 1A and fig. S1). To exclude confounding fluorescence by immune cells, αSMA-RFP/GFP mice were lethally irradiated and transplanted with the bone marrow from wild-type, nonfluorescent C57BL/6 donors (Fig. 1A and fig. S1). The resulting αSMA-RFP/GFP(stroma) reporter mouse displayed GFP- and RFP-expressing αSMA^+^ stromal cells, as detected by iMPM through a dorsal skinfold chamber (fig. S2). Before material implantation, the superficial interstitial connective tissue of the hypodermis was covered by a thin layer (~10 to 20 μm thick) of quiescent GFP^+^ αSMA-RFP^−^ cells displaying fibroblast features, including spread, stellar-like shape with lamellipodia (*27*). Deeper skin layers additionally contained adipocytes, nerves, and muscle fibers (fig. S2), with a limited number of GFP^+^ αSMA-RFP–expressing fibroblasts and pericytes. Besides fluorescent stromal cells, the tissue did not show evidence of infiltrating GFP^+^ immune cells (fig. S2). Implanted material consisted of porous scaffolds (5 mm × 5 mm × 0.25 mm), made of electrospun PCL, a hydrophobic and semicrystalline polymer prepared by ring opening of ε-caprolactone, and endowed with no positive or negative charge (*29*). After implantation, the recruitment and activation of GFP^+^ αSMA-RFP^−^ and GFP^+^ αSMA-RFP^+^ fibroblasts were monitored longitudinally for 11 days (Fig. 1, A to C). The total number of GFP^+^ cells significantly increased by day 7, reaching a plateau by day 11 (Fig. 1, B and D). Whereas directly after scaffold implantation resident stromal cells lacked αSMA expression, only 1 day later, αSMA-RFP expression was up-regulated in a subset of GFP^+^ cells (~10%; Fig. 1, B, C, and E). By day 7, the GFP^+^ αSMA-RFP^+^ subset increased to 50%, reaching ~100% by day 11 (Fig. 1, B, C, and E). Whereas the morphology of GFP^+^ αSMA-RFP^−^ resident cells was generally spread, the area covered by each fibroblast decreased by day 1 after implantation before αSMA up-regulation (Fig. 1, C and F). The observed shape change associated with αSMA induction is in line with the evolution of resting fibroblasts to protomyofibroblasts, which precedes αSMA expression and full maturation to contractile myofibroblasts (*30*). To examine whether the conversion from resting to αSMA^+^-activated fibroblasts modulates fibroblast migration, we performed time-resolved cell tracking at days 0, 4, 7, and 11 after PCL scaffold implantation. GFP^+^ αSMA-RFP^−^ cells (day 0) were positionally stable, with a dynamic cytoplasmic protrusive activity (Fig. 1, G and H, and fig. S3). By day 4, both GFP^+^ αSMA-RFP–negative and αSMA-RFP–positive cells increased their speed and invaded as individual elongated, spindle-shaped cells (movie S1; Fig. 1, G and H; and fig. S3). With an acceleration reaching 0.4 to 0.6 μm/min, this mesenchymal migration mode was retained in αSMA-RFP^+^ cells up to day 11. Thus, a migrating state emerges in fibroblasts before αSMA expression and persists in GFP^+^ αSMA-RFP^+^ cells. In our time-lapse recordings from day 4, cell division was a rare event (<1% over 4 hours) in both αSMA-RFP–negative and αSMA-RFP–positive cells (*n* = 2 dividing cells per subset in >300 recorded cells; movies S2 and S3). These results suggest that fibroblast activation and αSMA up-regulation are initiated within 24 hours after biomaterial implantation and concur with the induction of fibroblast migration rather than proliferation.

### Spatiotemporal 3D mapping of αSMA^+^ fibroblast activation and fibrosis

**Fig. 2.**
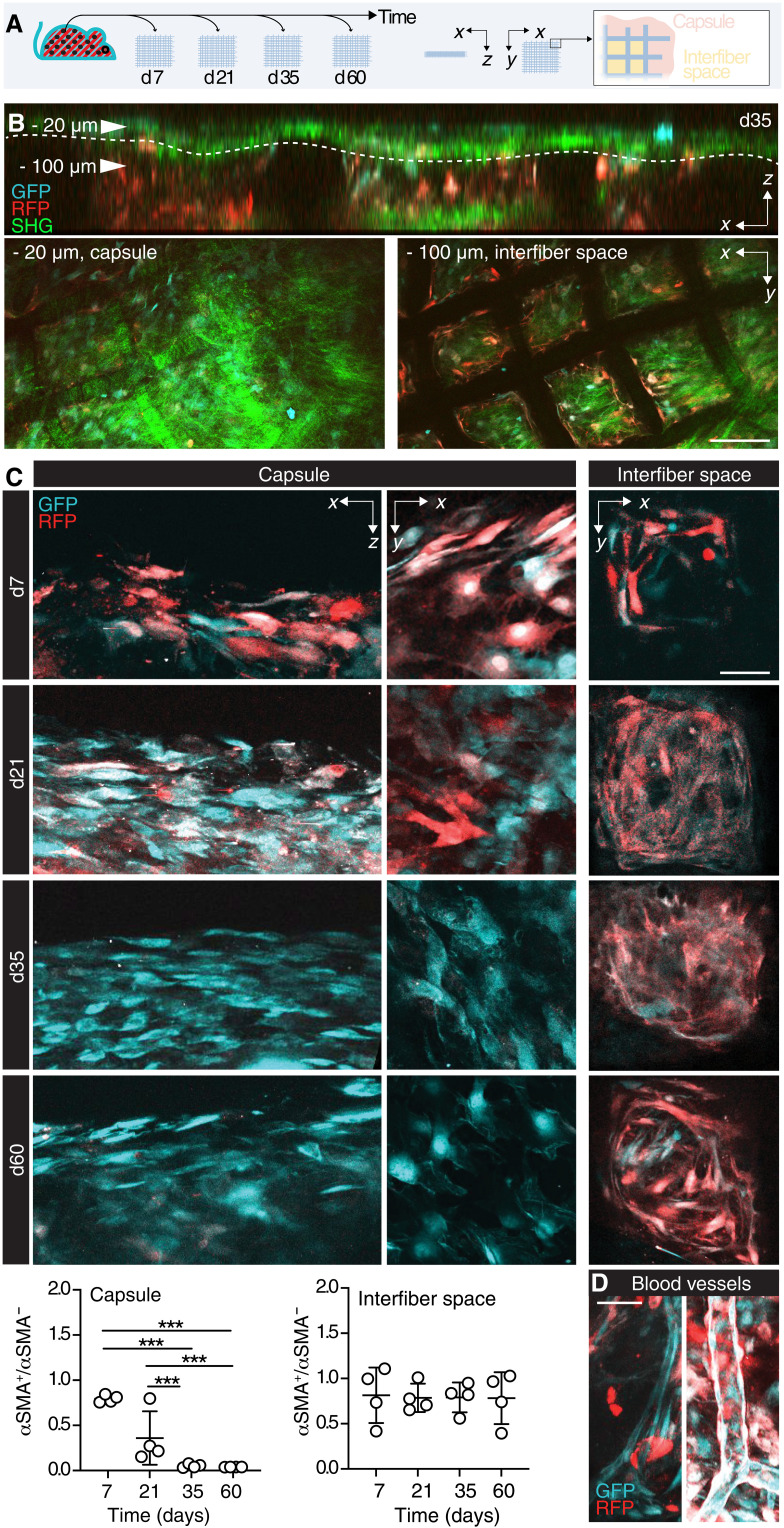
Longitudinal analysis of fibroblast activation. (**A**) Schematic representation of the model; PCL scaffolds were implanted subcutaneously in the back of αSMA-RFP/GFP(stroma) reporter mice (*n* = 4 per group); explanted at days 7, 21, 35, and 60; and analyzed by ex vivo MPM. (**B**) Orthogonal view and details of the capsule and interfiber space. Scale bar, 100 μm. (**C**) Representative images of the fibroblast activation in the external capsule (overviews shown as *xy* and *xz* sections) and inside the scaffold pores (XY sections, interfiber space), at days 7, 21, 35, and 60. Dot plots (ratio between the area covered by GFP and RFP signal) in the capsule and the interfiber space are shown over time. GFP^+^ cells, cyan; αSMA-RFP^+^ cells, red. Scale bar, 50 μm. (**D**) Details of blood vessels covered with RFP^+^ pericytes. Scale bar, 50 μm. GFP^+^ cells, cyan; αSMA-RFP^+^ cells, red; collagen, SHG, green. ***P < 0.001; one-way ANOVA followed by Tukey’s HSD post hoc test.

To identify the impact of the temporal progression and subregions of αSMA up-regulation, αSMA-RFP/GFP(stroma) mice implanted with PCL scaffolds were monitored by ex vivo 3D MPM at different time points (7, 21, 35, and 60 days after implantation; Fig. 2A). We identified two subregions: a fibrotic capsule, comprising fibroblasts and bundled collagen [detected by second harmonic generation (SHG)], which is connected to the surrounding interstitial tissue, and an inner core zone, where cells and collagen fill the space between implant structures (Fig. 2, A and B). By day 7 after implantation, 85% of the GFP^+^ cells also expressed αSMA-RFP, with comparable frequency in both subregions of the implant (Fig. 2C). By day 21, αSMA expression in the core, between PCL fibers, remained stable; however, it gradually declined in the capsule and returned to αSMA negativity by day 35. Notably, 60 days after implantation, αSMA-RFP^+^ cells persisted in the graft core, while no αSMA-RFP^+^ cell was visible within the fibrotic capsule surrounding the implant (Fig. 2C). On the basis of morphology and patterning, we excluded that the interfiber-activated cells were pericytes only (Fig. 2D), because rare blood vessels were covered by aligned αSMA^+^ cells (Fig. 2D). These results define the end stage of the FBR as a two-compartment process, consisting of an αSMA cold fibrotic capsule and an αSMA hot core with ongoing fibroblast activity.

### αSMA^+^ fibroblast self-organization and interaction with the implant

**Fig. 3.**
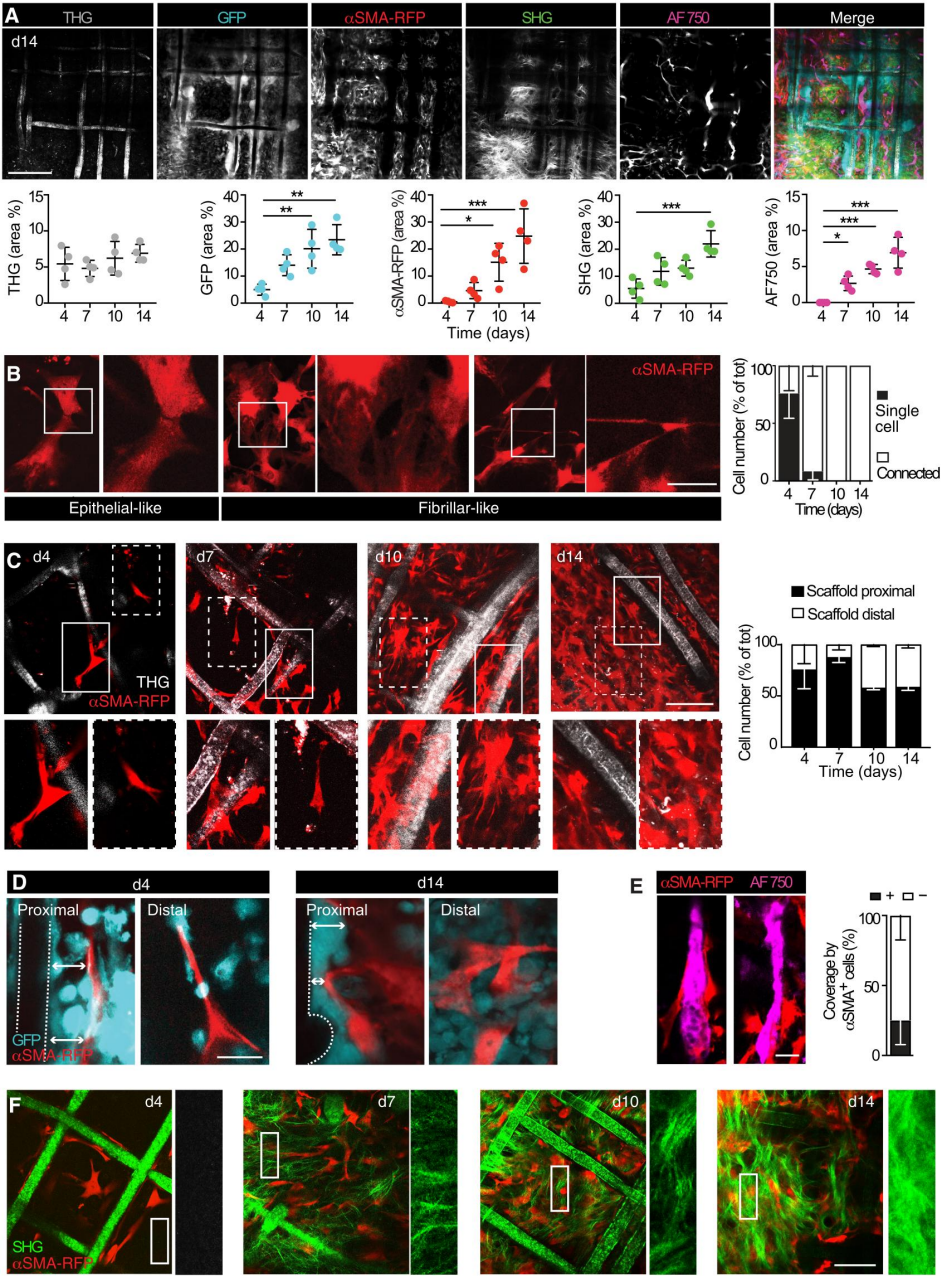
Longitudinal iMPM imaging of αSMA^+^ fibroblast interaction with partner elements of the FBR. (**A**) FBR at day 14 after implantation, single channels, and merge. Scale bar, 100 μm. A quantification is shown; AF750, Alexa Fluor 750 ; means ± SD, *n* = 4 mice per implant; four independent fields per implant were averaged. *P < 0.05, **P < 0.01, and ***P < 0.001; one-way ANOVA followed by Tukey’s HSD post hoc test. (**B**) Details of αSMA^+^ cell interactions captured by iMPM and quantification; three mice, two implants per mouse, two independent fields per implant. Box, inset; scale bar, 10 μm. (**C**) Kinetics of recruitment of αSMA^+^ cells in relation to the distance from the scaffold; a quantification is shown. Solid box, proximal cells; dashed box, distal cells. Magnifications are shown; two independent fields per implant, three mice, two implants per mouse. Scale bar, 100 μm. (**D**) Interaction of αSMA^+^ cells with GFP^+^ immune cells in regions proximal to the PCL fiber (proximal) or in the interfiber space (distal), 4 and 14 days after the scaffold implantation. Dotted line, scaffold; arrow, space between scaffold and αSMA^+^ cells. Scale bar, 20 μm. (**E**) Interaction of αSMA^+^ cells with neovessels (AF750). Histogram, percentage of vascular coverage by the αSMA^+^ cells, 14 days after the scaffold implantation. *n* = 3 mice, four areas per implant. Scale bar, 20 μm. (**F**) Collagen deposition. Merged representation of αSMA^+^ cells and SHG detection (scaffold fibers and the collagen bundles) over time. Magnifications of the SHG channel for each time point are shown. Scale bar, 100 μm.

To gain insight into the organization of αSMA^+^ cells during material encapsulation, their key homotypic and heterotypic interactions with cellular, extracellular, and noncellular elements of the FBR were monitored by longitudinal multiparametric iMPM. αSMA-RFP mice transplanted with the bone marrow of a GFP donor mouse were used (fig. S1), which allow reliable discrimination of myofibroblasts (GFP^−^ αSMA-RFP^+^) from inflammatory cells (GFP^+^; fig. S4, A to D). The resulting αSMA-RFP^GFP^ mouse showed fully reconstituted GFP^+^ bone marrow (fig. S4, B to D) and peripheral organs, such as the skin (fig. S5). Multiparameter image acquisition included the PCL implant [third harmonic generation (THG) and SHG], GFP immune cells, RFP-activated fibroblasts, perfused blood vessels (Alexa Fluor 750–labeled 70-kDa dextran), and fibrillar collagen (SHG). The implant site was progressively infiltrated by αSMA-RFP^+^ cells and GFP^+^ immune cells, paralleled by neoangiogenesis by day 7 and collagen deposition by day 14 (Fig. 3A and fig. S6), as described (*26*). Initially, αSMA-RFP^+^ fibroblasts were recruited as single cells and reciprocally interacted to form an interconnected network by day 4, with >90% of the cells connected by homotypic epithelial-like or fibrillar-like interactions by day 7 (Fig. 3B). Epithelial-like junctions were blunt and involved a broader area of contact, while fibrillar-like junctions were thin and elongated and could extend up to 20 μm to reach more distant cells (Fig. 3B). These connections are predicted to sense the reciprocal distribution in space and autoregulate the cellular density (*27*, *31*). To determine the positioning of αSMA-RFP^+^ fibroblasts relative to the scaffold fibers over time, we distinguished the proximal positioning of fibroblasts (within ≤50-μm distance from the PCL implant) from distal positioning (defined as >50 μm from the scaffold fiber) and counted them. At days 4 and 7 after implantation, αSMA^+^ fibroblasts preferentially distributed near PCL fibers rather than within the interfiber spaces, followed by equal distribution between the proximal and distal positioning by day 10 (Fig. 3C). This may suggest that connective tissue formation initiates from the solid scaffold structures toward free space. Notably, neither at the early nor late stages of the FBR, αSMA^+^ fibroblasts were detected in direct contact with the PCL material (Fig. 3D). This spatial separation was maintained by immune GFP^+^ cells that are recruited to the scaffold fibers and mature to FBGCs (*26*). These infiltrating cells were expressing interferon regulatory factor 5 (IRF-5), a marker of the M1 phenotype (fig. S8), which is typically associated with a proinflammatory activity, and lacked the M2 marker CD163, which is expressed by immunomodulatory macrophages (*32*, *33*). αSMA^+^ cells further interacted with blood vessels, covering about 30% of the material-induced vasculature (Fig. 3E). Despite early onset of fibroblast activation, significant de novo collagen deposition initiated only between days 4 and 7 after implantation (Fig. 3F). This is consistent with the concept of two functional types of αSMA^+^ fibroblasts, including a collagen nonsecretory and a secretory populations (*30*). In conclusion, without directly engaging with the material, αSMA^+^ fibroblasts first populate the implant site as nonsecretory cells and establish a dynamic multicellular network during the phase of collagen secretion.

### αSMA^+^ fibroblast activation in relation to cellular subcompartments

**Fig. 4.**
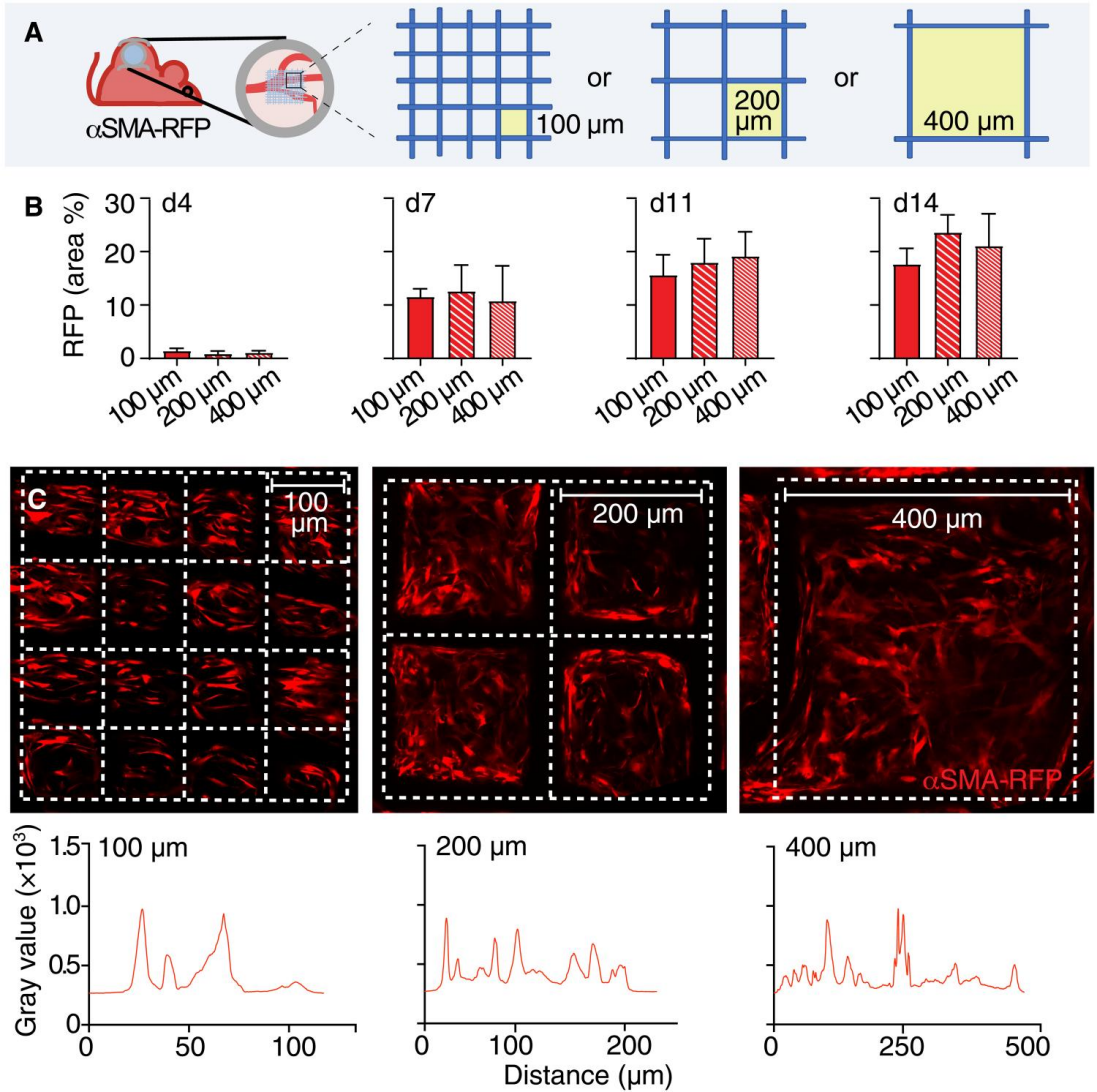
Analysis of αSMA-RFP^+^ cell recruitment by scaffolds of different grid sizes. (**A**) Schematic representation of the model; scaffolds of three different grid sizes (pore sizes of 100 × 100, 200 × 200, and 400 × 400 μm) were implanted inside the DSFC. (**B**) Total amount of αSMA-RFP^+^ cells recruited measured as % area occupied, over time (days 4, 7, 11, and 14). (**C**) Overviews of the fibroblast distribution in the three different scaffold types. Dotted lines, scaffold position. XY intensity profile along single pores for each of the three scaffold geometries is shown.

To further identify whether the presence of cellular subcompartments, such as enrichment of the specialized FBGCs, affects the recruitment and positioning of αSMA^+^ cells, we implanted PCL scaffolds with varying pore sizes (100 × 100, 200 × 200, or 400 × 400 μm grid) and monitored the recruitment and self-organization αSMA^+^ cells over time (Fig. 4A). The grid size and relative different density of FBGCs did not affect the kinetics of αSMA^+^ cell recruitment at any time point (Fig. 4B). Furthermore, the spatial analysis did not show preferential proximal or distal positioning relative to the scaffold fibers (Fig. 4C). Thus, implantation of a PCL scaffold, regardless of its porosity, was sufficient to induce recruitment, activation, and redistribution of αSMA^+^ cells, which equally distributed regardless of the distance from the material interface and FBGCs.

### Effects of macrophage ablation on αSMA^+^ fibroblast recruitment and positioning

**Fig. 5.**
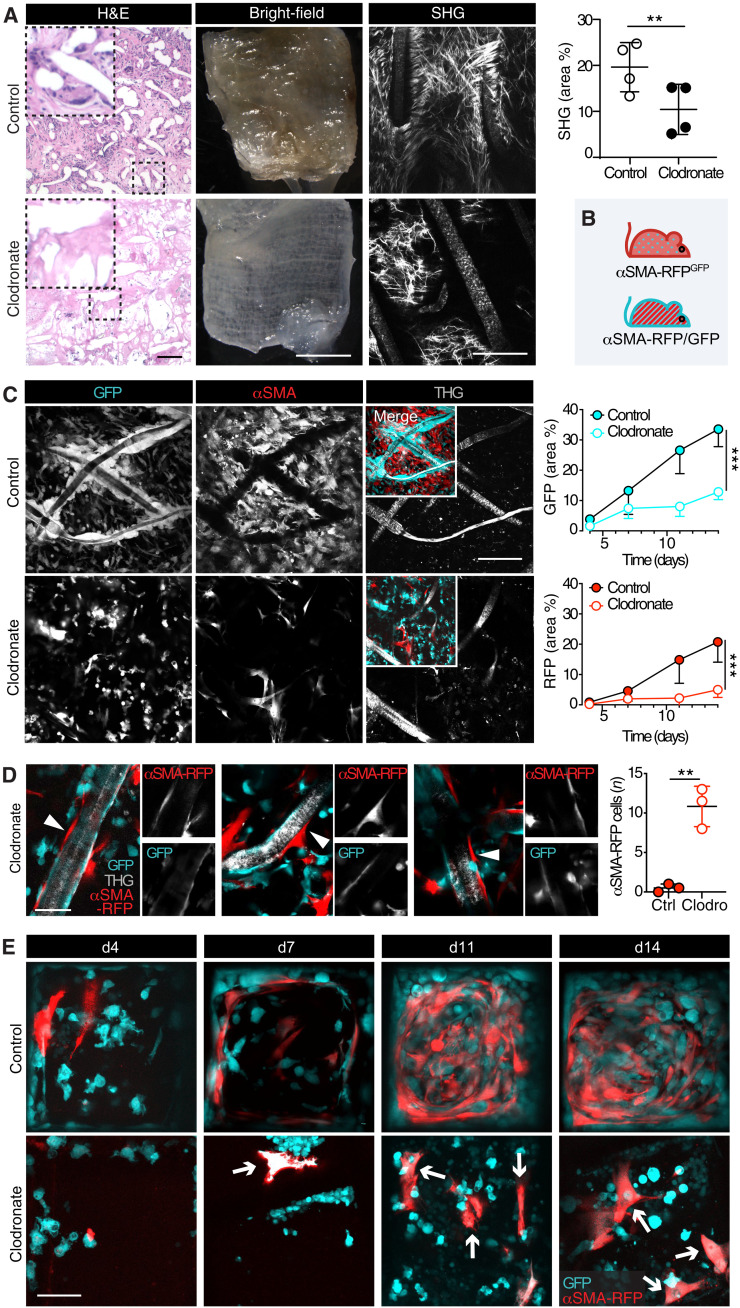
Monitoring the fibrosis-driving macrophage-fibroblast cross-talk in αSMA-RFP/GFP mice. (**A**) Histology (H&E, hematoxylin and eosin ; left), bright-field, and SHG microscopy in control- and clodronate liposome–treated whole-mount samples. Bright-field overviews of PCL scaffolds day 14 after implantation in control- or clodronate liposome–treated mice. Zooms show collagen fibers associated with PCL fibers. Scale bar, 1 mm (overviews) and 100 μm (zooms). Images were acquired ex vivo by multiphoton microscopy. Scale bar, 100 μm. Quantification of the area covered by the SHG signal (means ± SD) for *n* = 4 scaffolds, one scaffold per mouse. **P < 0.01; unpaired two-tailed Student’s *t* test. (**B**) Cartoon, mouse models applied for clodronate treatment. (**C**) Longitudinal iMPM of the FBR elicited by PCL in control- or clodronate liposome–treated αSMA-RFP^GFP^ mice after scaffold implantation. Micrographs represent single and merged channels at day 14 after implantation. Scale bar, 100 μm. Graphs show the means ± SD of GFP- and RFP-covered area for PCL in control- and liposome-treated mice (*n* = 4 mice, one implant per mouse; two independent fields per implant were averaged). ***P < 0.001; unpaired two-tailed Student’s *t* test. (**D**) Effect of clodronate on cell recruitment and topology of fibroblasts and infiltrating cells (day 14, three independent examples are shown). White arrowhead, fibroblast in direct contact with the material. Scale bar, 50 μm. The graph shows the means ± SD of the number of αSMA-RFP^+^ cells within 5 μm of distance from the scaffold (*n* = 3 mice, one implant per mouse; two independent fields per implant were averaged). **P < 0.01; unpaired two-tailed Student’s *t* test. (**E**) Activation of αSMA-RFP^+^ cells (red) in αSMA-RFP/GFP in control- and clodronate liposome–treated dual-color mice, over time (*n* = 4 mice, one implant per mouse). GFP^+^ cells, cyan; arrow, αSMA-RFP^+^ cells. Scale bar, 50 μm.

Macrophages and FBGCs, two key regulators of the FBR, actively support local fibrotic encapsulation (*8*, *26*). To address whether αSMA induction and myofibroblast network formation are orchestrated by myeloid cells, we depleted the macrophage lineage in scaffold-bearing mice using clodronate liposomes. Clodronate, a first-generation bisphosphonate that causes apoptosis upon internalization within phagosomes (*34*), ablated macrophages and FBGCs, followed by markedly reduced collagen deposition and fibrotic encapsulation of the implant (Fig. 5A). When administered to mouse fibroblasts (NIH 3T3 cells), in vitro, clodronate liposomes did not affect their viability (fig. S7), as previously shown for smooth muscle and endothelial cells (*35*). As monitored by longitudinal iMPM over 14 days, clodronate-treated αSMA-RFP^GFP^ mice (Fig. 5B and fig. S1) showed a significantly decreased infiltration of GFP^+^ immune cells (60% less) and lacked FBGCs, compared to control mice (Fig. 5C). The decrease of infiltrating immune cells was paralleled by a 75% reduction of the αSMA-RFP^+^ population (Fig. 5C). Likewise, αSMA-RFP^+^ fibroblast network formation was prevented, with spare connections formed between individual cells (Fig. 5C). Whereas in control mice direct interaction of αSMA^+^ fibroblasts with the polymer was usually prevented by the physical interpositioning by FBGCs, clodronate treatment allowed direct interaction with the PCL surface and overall recruitment of a significantly higher number of αSMA^+^ fibroblasts within 5 μm of distance from PCL fibers (Fig. 5, C and D). Thus, both multicellular self-organization to networks and interstitial positioning without direct contact with the implant material were perturbed by the ablation of macrophages. As a limitation, αSMA-RFP^GFP^ mice displayed fluorescent αSMA^+^ fibroblasts only (fig. S1), so we could not exclude the presence of αSMA^−^ fibroblasts. To monitor whether the activation of αSMA was affected by clodronate treatment, αSMA-RFP/GFP dual-color mice were treated with clodronate liposomes. In these mice, all cells express GFP, including immune cells (which we monitored as a readout of clodronate inhibition), and fibroblasts, with αSMA^+^ cells additionally up-regulating RFP (Fig. 5B and fig. S1). All the fibroblasts recruited by scaffolds implanted in mice treated with clodronate liposomes displayed αSMA expression, whereas GFP^+^ αSMA^−^ spindle-shaped multicellular networks were not detected (Fig. 5E), which suggests that conversion to αSMA^+^ state is unperturbed in the fibroblast subset. In conclusion, clodronate treatment reduced the recruitment of infiltrating immune cells, as expected, and decreased the number of αSMA^+^ cells and their positioning but did not impair their activation.

### αSMA^+^ fibroblast engagement in response to the material composition

**Fig. 6.**
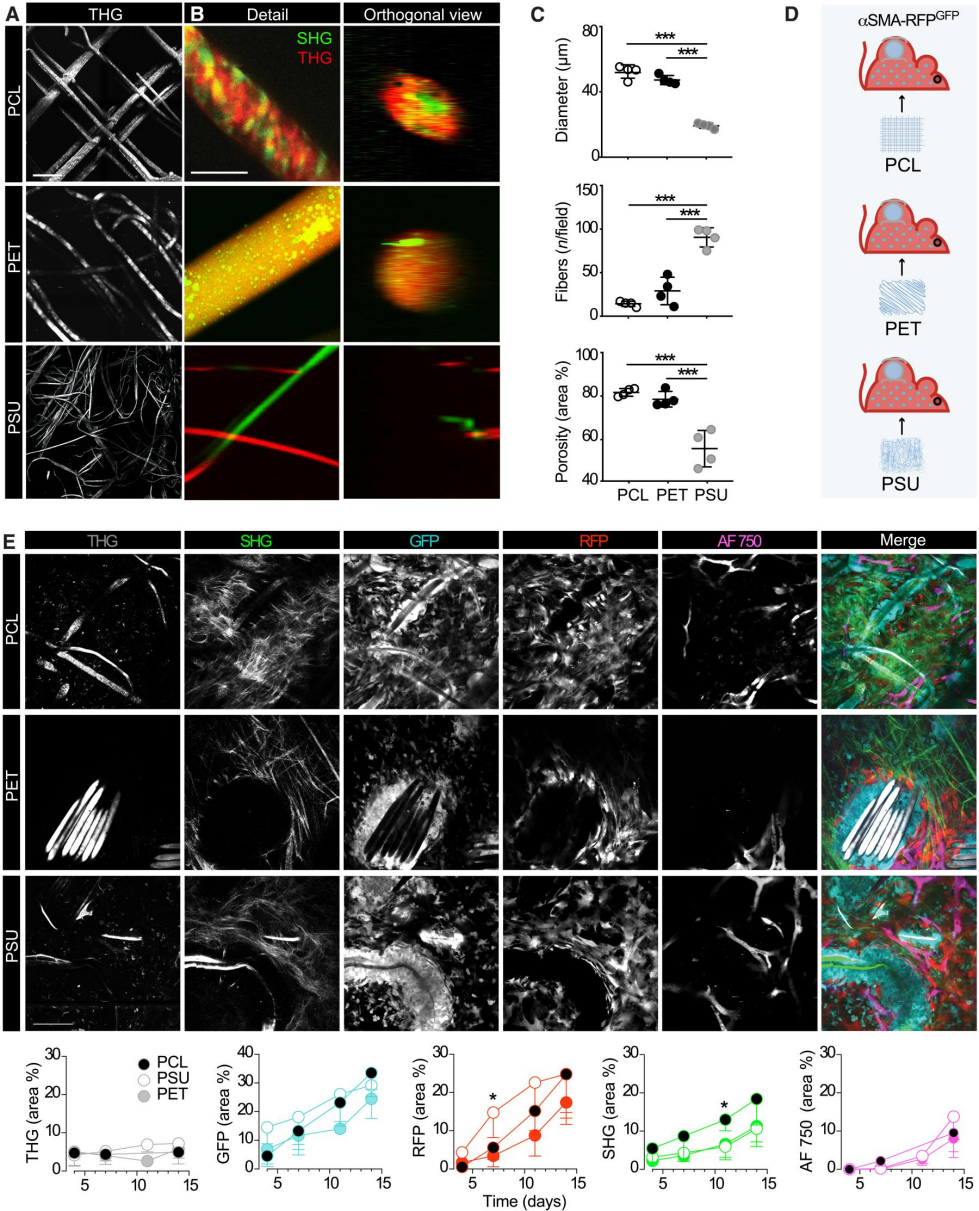
Longitudinal intravital imaging of the FBR in the three different biomaterials. (**A**) 3D reconstruction of PCL, PSU, and PET scaffolds by THG, macroscopic overview; scale bar, 100 μm. (**B**) High-resolution SHG and THG projection of a single fiber of each scaffold in the horizontal (*xy*) and orthogonal (*xz*) directions. Scale bar, 50 μm. (**C**) Quantification of fibers diameter, number of fibers per field, and porosity of each scaffold (image size, 360 × 360 μm) are shown, means ± SD.***P < 0.001 by one-way ANOVA followed by Tukey’s HSD post hoc test. (**D**) Schematic representation of the model; PCL, PET, or PSU scaffolds were implanted subcutaneously in the back of an αSMA-RFP^GFP^ reporter mouse analyzed by iMPM. (**E**) Longitudinal iMPM of the FBR elicited by different biomaterials (PCL, PET, and PSU). Single channels and merged representations at day 14 after implantation are shown. THG (gray); GFP-positive cells (cyan); GFP-positive cells (red); Alexa Fluor 750 70-kDa dextran (magenta) and SHG (green). Scale bar, 100 μm. Bottom panels, a quantification of THG, SHG, GFP, RFP, and Alexa Fluor 750 for the different biomaterials at days 4, 7, 10, and 14 are shown; means + SD. *n* = 4 mice per implant; four independent fields per implant were averaged. *P < 0.05 by one-way ANOVA followed by Tukey’s HSD post hoc test. No significant differences were identified at day 14 after implantation in any of the parameters detected, as analyzed by one-way ANOVA followed by Tukey’s HSD post hoc test. Scale bar, 100 μm.

Material properties, such as composition, charge, and porosity, can modulate the severity of the FBR (*1*, *6*, *36*). Thus, we hypothesized that modulating the material type and geometry of the implant would also affect the recruitment and self-organization of αSMA^+^ cells. Therefore, we compared the kinetics and extent of the FBR after implantation of PCL or two clinically relevant porous biomaterials, polysulfone (PSU) and polyethylene terephthalate (PET). PSU is a rigid and amorphous thermoplastic polymer constituted by aromatic groups, joined by a sulfone group, which displays a weakly negative charge (*37*). PET, the most common thermoplastic polymer of the polyester family, consists of polymerized units of the monomer ethylene terephthalate; it may exist as an amorphous or semicrystalline polymer, with a negative surface charge (*38*, *39*). Geometrically, PCL and PET scaffolds were similar in fiber diameter, number of fibers, and porosity, while the PSU fiber diameter was significantly smaller and the fiber density was higher (Fig. 6, A to C). When longitudinally monitored by iMPM (Fig. 6D), the three different implants became gradually infiltrated by GFP^+^ and αSMA-RFP^+^ cells, followed by deposition of fibrillar collagen and perfused neovessels (Fig. 6E) in line with the previous analysis of PCL (Fig. 3). PSU caused an accelerated emergence of αSMA^+^ fibroblasts compared to PCL and PET, followed by comparable levels of both cell subsets by day 14 (Fig. 6E). Whereas collagen deposition showed an accelerated trend in response to PCL, despite delayed αSMA-RFP^+^ cell recruitment, collagen content by day 14 did not differ among implant types. Neovascularization was not statistically different in the biomaterials tested (Fig. 6E). We further examined whether these biomaterials elicit macrophage polarization to a different extent (figs. S8 and S9) by detecting the expression of IRF-5 and CD163, two established markers of M1 and M2 polarization that we previously applied (*26*, *40*–*42*). PCL induced an M1-dominated response in macrophages and FBGCs, with very rare M2-infiltrating macrophages and no M2-positive FBGCs at day 14 (figs. S8 and S9), whereas PSU and PET elicited a mixed M1/M2 response (fig. S9). Notably, in contrast to macrophages and regardless of the implanted biomaterial, FBCGs developed a uniform M1 polarization (fig. S9). Thus, porous materials that differed (*43*) in composition, charge, geometry, and M1/M2 polarization capability induced a highly conserved program of activation and self-organization of αSMA^+^ fibroblasts in coordination with M1-type polarization of immune infiltrating cells.

## DISCUSSION

Fibroblasts are known effectors of fibrosis in the FBR, but their recruitment, activation, and dependence on myeloid-derived cells remain mostly unexplored (*13*). We here close a knowledge gap by applying iMPM in situ to dissect the contribution of fibroblasts in promoting the FBR and identify their step-wise activation and self-organization as a canonical conserved process that is partially independent of macrophage-derived signals.

### Timing of self-organization to a secretory fibroblast network

Mouse fibroblasts are recruited in early granulation tissue 2 to 4 days after wounding (*44*, *45*). In the FBR, fibroblasts infiltrated polymeric porous materials as early as 1 to 4 days after implantation. However, instead of a multicellular, dense front of an activated fibroblast network collectively invading an acute surgical wound (*46*), initial recruitment occurred as sparsely and evenly distributed single fibroblasts, which dynamically self-assembled in a multicellular network by day 7. This is consistent with fibroblasts reciprocally sensing their distribution in space through interconnectivity by filamentous protrusions, which allows for autoregulating their cellular density (*27*, *31*). As a key difference, the healing of an excisional wound aims to rapidly adapt the margins. During the FBR, instead, the wounded tissue hosts a cell- and collagen-free material that is filled by a provisional fibrin and plasma protein network and gets progressively populated by individually immigrating immune and stromal cells, which self-assemble to form a de novo desmoplastic tissue. These data suggest that networks can form via two distinct kinetic mechanisms in response to the type of tissue damage: self-assembly from individually immigrated fibroblasts or network invasion.

### αSMA activation

αSMA activation occurred as soon as 1 day after implantation in 10% of the cells, with widespread expression within 10 days. This timing of fibroblast activation is consistent with the kinetics of fibroblast activation achieved for deep fascia wound healing (*46*, *47*). The expression of αSMA, which confers high contractile force via stress fibers (*19*, *30*), was correlated with a progressive reduction of cell spreading. This in vivo outcome is in line with the gradual maturation process that leads resting fibroblasts to evolve into contractile αSMA^−^ protomyofibroblasts, followed by maturation into highly contractile αSMA^+^ myofibroblasts (*19*, *30*). Throughout all phases monitored by iMPM, both αSMA^−^ and αSMA^+^ cells showed limited proliferative ability, suggesting that they were likely engaged in the process through migration. We could not exclude recruitment of αSMA^+^ fibroblasts from intact deeper skin layers due to insufficient *z*-resolution of iMPM precluding optical access to tissue below the implant.

### Identification of a long-term activation niche

Long-term imaging of wound healing processes has shown a recession of myofibroblasts by day 70 after injury (*47*). After implantation of porous materials, the αSMA activation program by fibroblasts was sustained up to day 60 within the inner core zone, where cells and collagen fill the interfiber space, while αSMA down-regulation occurred in fibroblasts localized in the external fibrotic capsule, which was completed after 35 days. As an outcome, the FBR to encapsulated porous material maintains two distinct niches: an internal αSMA^+^ hot environment confined inside a peripheral αSMA^−^ cold capsule. The nature of these two niches requires further exploration to understand the determinants of persistent fibroblast activation and inactivation, which might be aimed at concealing the presence of an active fibrotic environment in the host. The presence of a relatively limited number of infiltrating immune cells within the fibrotic capsule and the potential relatively lower stiffness/tension of this surrounding fibrotic tissue (compared to the polymeric material) could play a role in supporting long-term αSMA activation. Accordingly, tensile forces exerted within microtissues in vitro have been shown to support the transition from fibroblasts to myofibroblasts and αSMA expression at the highly tensed growth front (*48*). Thus, although the quiescent fibrous capsule surrounding the implant may suggest an inert, shielded integration, the persistent αSMA activation may sustain the activity of the FBR from inner implant regions, notunlike a wound that never heals. Such persistent αSMA expression could offer an opportunity for targeted therapies. This includes chimeric antigen receptor–expressing T cells (*49*, *50*) and anti-TGFβ signaling strategies, although the latter has proved challenging, with limited/no efficacy in patients and causes adverse effects possibly linked to a broad inhibition of the homeostatic responses (*51*, *52*).

### Fibroblast heterogeneity

Fibroblasts present distinct molecular phenotypes based on their organ of origin and spatial location (*15*), but the subset heterogeneity in the FBR is overall not well characterized. Several markers have been used to classify fibroblasts, including αSMA, fibroblast activation protein (FAP), PDGF receptor-α and PDGF receptor-β (PDGFRβ), fibroblast-specific protein 1 (FSP1), vimentin, desmin, and discoidin domain-containing receptor 2 (*14*). However, none of these markers are specific for myofibroblasts: FSP1 is also expressed in macrophages and cancer cells, FAP is expressed in some immune cells, and desmin and PDGFRβ are in perivascular cells (*14*). In addition, the level of expression of putative markers varies greatly at the single-cell level, which confounds reliable subset classification. Recently, single-cell RNA sequencing has allowed capturing fibroblast heterogeneity in both healthy tissues and pathological conditions, including fibrotic diseases, cancers, and wound healing (*53*–*56*). This work has identified multiple fibroblast subtypes characterized by high transcriptional heterogeneity and different gene expression signatures. To more extensively characterize the status of quiescent and activated fibroblasts in the FBR, future studies will be required to identify the temporal and spatial evolution of fibroblast subsets and their roles in the perpetuation of the FBR and fibrosis.

### Regulation of the FBR by macrophages

The macrophage lineage has been established as a key driver of the FBR and fibrosis (*4*, *6*, *8*, *12*). However, in response to the implantation of porous polymeric materials, comparable αSMA^+^ cell recruitment, fibroblast network assembly, and collagen production were obtained regardless of M1 (PCL) or mixed M1/M2 infiltrate (PSU; PET). This M1/M2 classification, which has been extensively applied in materials science (*1*, *12*, *33*, *57*), may oversimplify the diversity of myeloid-derived cell activation and requires further investigation on the differential impact on the step-wise myofibroblast recruitment and activation in response to a greater variety of materials.

The elimination of the macrophage population by clodronate administration affected αSMA^+^ cell absolute number (that was reduced by ~75% but not totally ablated), their positioning (which usually lacks direct contact of fibroblasts with the material), and the density required to self-organize into a mature interconnected network but did not perturb αSMA expression. The remaining αSMA^+^ myofibroblasts were still able to generate a de novo fibrotic matrix, albeit at much-reduced overall density and network formation, suggesting that their principal ability to deposit collagen fibers was unperturbed. Macrophage-independent regulation of fibrosis may include molecular cues derived from other immune cells [e.g., B cells (*1*)], endothelial cells, or autocrine stimulation. In addition, high strain/mechanical stress induced by material implantation and implant stiffness can further modulate myofibroblast activation without affecting the number of macrophages (*58*).

### Material properties and antifibrotic effects

Antifibrotic materials generally focus on attenuating inflammation and consequently fibrosis (*1*). Recent studies, however, suggest that decreased levels of fibrosis might result from direct modulation of fibroblast activation. Modifications of silicone implant surface topography (average roughness, 0 to 90 μm) influenced the overall establishment of material-induced fibrosis, with an average surface roughness of 4 μm eliciting the least amount of FBR (*36*). In addition, materials printed with a micrometer-scale hexagonal pattern have shown antiadhesive properties by lowering myofibroblast activation and spreading (*59*). Softening of the material surface prevents stiffness-mediated fibroblast activation of implants, in vitro and in vivo (*58*, *60*, *61*), and similar results have been achieved in vitro through hydrogel coating, which decreased myofibroblast adhesion possibly because of higher hydrophilicity and lowered stiffness (*62*). Follow-up studies directly comparing materials with varying topography and stiffness will allow discrimination of how material properties stimulate fibroblast recruitment, dynamics, and self-organization.

In conclusion, αSMA-expressing fibroblasts are a persistent element of the FBR. Their recruitment, activation, and self-organization results in a conserved two-compartment process irrespective of the type of porous implant material, which might have major implications for perpetuating the fibrotic status.

## MATERIALS AND METHODS

### Scaffold design and fabrication

PCL scaffolds were produced by the Biomaterials Lab, Rice University, Houston, TX, USA; PSU scaffolds were from eSpin Technologies, Chattanooga, TN, USA; PET hernia repair meshes were from Medtronic, Dublin, Ireland. To fabricate scaffolds, PCL (43 kDa, Polysciences; Warrington, PA) was melted at 85°C and printed at a collector velocity of 40 mm s^−1^, 5.0 kV, 1.0 bar, and at a distance of 10 mm using a 3D Discovery Evolution printer, RegenHU, Switzerland. Scaffolds, designed using computer-aided design software BioCAD (RegenHU, Switzerland), had a filament width of 40 μm and a pore size of 100 × 100, 200 × 200, or 400 × 400 μm.

### Mouse model generation

Animal studies were approved by the Institutional Animal Care and Use Committee of The University of Texas, MD Anderson Cancer Center, which is accredited by the Association for Assessment and Accreditation of Laboratory Animal Care. Mice were housed with a maximum of five animals per cage in a state-of-the-art, air-conditioned, specific pathogen–free animal facility, and all procedures were performed in accordance with the National Institutes of Health (NIH) Policy on Humane Care and Use of Laboratory Animals. C57BL/6 wild-type mice were from the Department of Experimental Radiology, UT MD Anderson Cancer Center. C57BL/6-Tg (UBC-GFP) 30Scha/J mice, which ubiquitously express GFP, were from the Jackson Laboratory. C57BL/6-Tg(Acta2-DsRed)1Rkl/J mice, which express DsRed RFP under the αSMA, were a gift of R. Kalluri, UT MD Anderson Cancer Center (*63*, *64*). C57BL/6-Tg (UBC-GFP) 30Scha/J and Tg(Acta2-DsRed)1Rkl/J mice were crossed to generate a dual-color model (αSMA-RFP/GFP mouse) that displays GFP in each cell and RFP in αSMA-expressing cells. Then, we established a mouse model that displays GFP in immune cells only and RFP in αSMA-expressing cells (αSMA-RFP^GFP^ mouse) by bone marrow transplantation, as previously described (*65*). Briefly, whole-body irradiation of recipient mice in a cobalt irradiator (Shepherd *Mark* I-65 137Cs γ irradiator) at 5.5 grays (Gy) was performed twice, with a recovery interval of 3 hours between doses, for a total of 11 Gy per mouse. Fresh bone marrow was flushed from the posterior leg bones of the GFP donor mice (femur and tibia) using ~1 ml of 2% fetal bovine serum in phosphate-buffered saline (PBS). The bone marrow cells were centrifuged for 5 min at 1500 rpm, the supernatant was discarded, and the red blood cells were lysated, adding 250 μl of water for 20 s. The cells were centrifuged again for 5 min at 1500 rpm, the supernatant was discarded, and PBS was added to obtain a cell suspension of 5 × 10^7^ to 10 × 10^7^ cells/ml. Each recipient mouse was infused with 100 μl of cell suspension by retro-orbital injection. We established a second bone marrow–transplanted mouse model (αSMA-RFP/GFP stroma) that displays nonimmune (fibroblasts, pericytes, muscle fibers, and nerves) GFP stromal cells and RFP αSMA-expressing cells. C57BL/6-Tg (UBC-GFP) 30Scha/J and Tg(Acta2-DsRed)1Rkl mice were crossed to generate the dual-color αSMA-RFP/GFP mouse, which was transplanted with the bone marrow–derived from wild-type C57BL/6 donor, following the procedure detailed above.

### Dorsal skinfold chamber model and scaffold implantation

Scaffolds were implanted within a dorsal skinfold chamber system or subcutaneously into >8-week-old mice, as previously described (*26*). Briefly, during surgery, a 5 mm by 5 mm by 0.25 mm scaffold was surgically implanted into the subcutaneous tissue of either imaging window-bearing or window-free mice. For surgery, mice were anesthetized using isoflurane, and buprenorphine (0.01 mg/kg) was given postoperatively through intramuscular injection.

### Image acquisition

For intravital microscopy, experiments were performed as previously described (*26*). Mice were anesthetized with isoflurane and stably mounted onto a temperature-controlled platform (37°C). The FBR elicited by an implanted scaffold was monitored using a custom intravital multiphoton microscope (LaVision BioTech) with three Ti:Sapphire lasers (Chameleon-XR, Coherent) and two optical parametric oscillators (APE/Coherent), resulting in a tunable excitation range from 800 to 1300 nm. Multispectral detection was performed using up to five backward or two forward photomultipliers and up to three excitation wavelengths in two consecutive scans, to separate the following excitation and emission channels: GFP (920 nm; 525/50 nm), DsRed (1090 nm; 595/40 nm), SHG (1090 nm; 525/50 nm), THG (1180 nm; 387/15 nm), and Alexa Fluor 750 (1180 nm; 810/90 nm). For intravital detection, long-working distance × 16 numerical aperture (NA) 0.8 water or × 25 NA 1.05 multi-immersion oil/water objectives (Olympus) were used. The volumes acquired were characterized by the same constant in-plane physical spatial resolutions of 360 × 360 μm, 1064 × 1064 pixels, while the depth physical resolution, in-between slices, was 5 μm, for a maximum depth of 250 to 300 μm. Images were acquired in a random fashion within the subcutaneous tissue up to the dermis. Perfused blood vessels were visualized by intravenous injection of Alexa Fluor 750–conjugated dextran (70 kDa; Invitrogen; 1 mg per mouse). mPCL-CaP, PSU, and PET scaffolds in vitro were analyzed using SHG and THG imaging.

### Digital image processing, segmentation, and quantitative analysis

Methods developed previously were further implemented for these analyses (*26*). Images were reconstructed, stitched, and analyzed using FIJI (*66*). Individual 3D scan fields representing *z*-projections of 50 to 300 μm were stitched to large-field montages for both overview and detailed analysis.

#### Area analysis

Quantitative analysis of THG, SHG, and fluorescent channels was performed on 3D stacks of 360 μm by 360 μm, with 10-μm step intervals in the *z-*direction. Single-channel *z*-stacks were masked, thresholded (default or Li algorithm), and converted to binary images; the signal-positive area was obtained and reported as percentage of the total area analyzed. For each sample, the relative fluorescence density was obtained from 10 slices per *z*-stack, averaged, and represented as the percentage of the total area. *n* = 4 mice per implant; four independent fields per implant were averaged. The experiment was repeated three times.

#### Quantification of αSMA-RFP^+^ cells

The number of αSMA^+^ cells next to the scaffold fiber or in the interfiber space, as well as the number of single versus connected αSMA^+^ cells, was manually quantified over time (days 4, 7, 11, and 14 after the scaffold implantation) using the support of the Cell Counter plugin (Kurt De Vos University of Sheffield, Academic Neurology) of ImageJ (NIH) (three mice, two implants per mouse, two independent fields per implant). The total number of GFP^+^ and αSMA-RFP^+^ cells over time (days 0, 1, 4, 7, and 10 after scaffold implantation) was automatically quantified using the HK-mean segmentation method of the image analysis platform Icy (*67*) (360 μm by 360 μm by 50 μm, three mice per time point).

#### αSMA^+^ cell size analysis

The perimeter of 50 random cells per time point (three mice) in the αSMA-RFP/GFP stroma mouse model was manually traced, and the area was automatically calculated.

#### Time-lapse analysis

The manual tracking (Cordelières F, Institut Curie, Orsay France) of ImageJ was used to record single-cell velocities from time-lapse sequences.

### Therapeutic intervention

Mice bearing the scaffolds in the subcutaneous tissue received clodronate liposomes (200 μl per mouse) every 2 to 3 days, starting 3 days before scaffold implantation to deplete macrophages by the day of implantation. For intravital microscopy, *n* = 4 mice per implant; two independent fields per implant were averaged. The experiment was repeated twice.

### Histological analysis

This analysis was performed as previously described (*26*). Briefly, mice were euthanized 14 days after implantation of the scaffold. Scaffold-bearing skin was excised, fixed (4% buffered formaldehyde), and embedded in paraffin for hematoxylin and eosin or IRF-5 and CD163 staining (five sections per sample, 5 μm thick, three to four samples per scaffold). The experiment was repeated twice.

### Cell culture

NIH 3T3 mouse fibroblasts were from the American Type Culture Collection. Cells were cultured in Dulbecco’s modified Eagle’s medium (Invitrogen) supplemented with 10% fetal bovine serum (Sigma-Aldrich) and 1% penicillin and streptomycin (both 100 μg/ml; Sigma-Aldrich). NIH 3T3 cells were seeded in a 96-well plate (*n* = 4 wells per group, 25,000 cells per well); 24 hours after, cells were treated for 48 hours with clodronate liposomes (1:100 and 1:200 of the original suspension) diluted in complete cell culture medium. Cell viability was measured by staining cells for 15 min with propidium iodide (PI; 25 μg/ml) and Hoechst (50 μg/ml) and by subsequent imaging using the EVOS FL Cell Imaging System (AMG) equipped with a 4× objective. The number of Hoechst^+^ and PI^+^ nuclei was counted using the Stardist plugin (*68*) in FIJI software, and the number of PI^+^ cells is expressed as a percentage of the total number of cells.

### Statistical analysis

For statistical analyses, Student’s *t* test was used for paired samples with Gaussian distribution. For independent samples, irrespective of distribution, the one-way analysis of variance (ANOVA) test was used. For all multiple analyses, Tukey’s honestly significantly different post hoc correction was performed. GraphPad Prism 9.2.0 software was used for statistical analysis.
