# Supplementary material for: Dissecting the recruitment and self-organization of αSMA-positive fibroblasts in the foreign body response
Source: Sci Adv. 2022 Dec 21;8(51):eadd0014. doi: 10.1126/sciadv.add0014 (PMC9770965; doi:10.1126/sciadv.add0014)
Supplement: Supplementary file 1 — Figs. S1 to S9 [file sciadv.add0014_sm.pdf]

Science Advances
AAAS

Supplementary Materials for

Dissecting the recruitment and self-organization of αSMA-positive fibroblasts in the foreign body response

Maria Parlani *et al.*

Corresponding author: Eleonora Dondossola, edondossola@mdanderson.org

Sci. Adv. **8**, eadd0014 (2022)
DOI: 10.1126/sciadv.add0014

The PDF file includes:

Figs. S1 to S9

Other Supplementary Material for this manuscript includes the following:

Movies S1 to S3

Supplementary figures

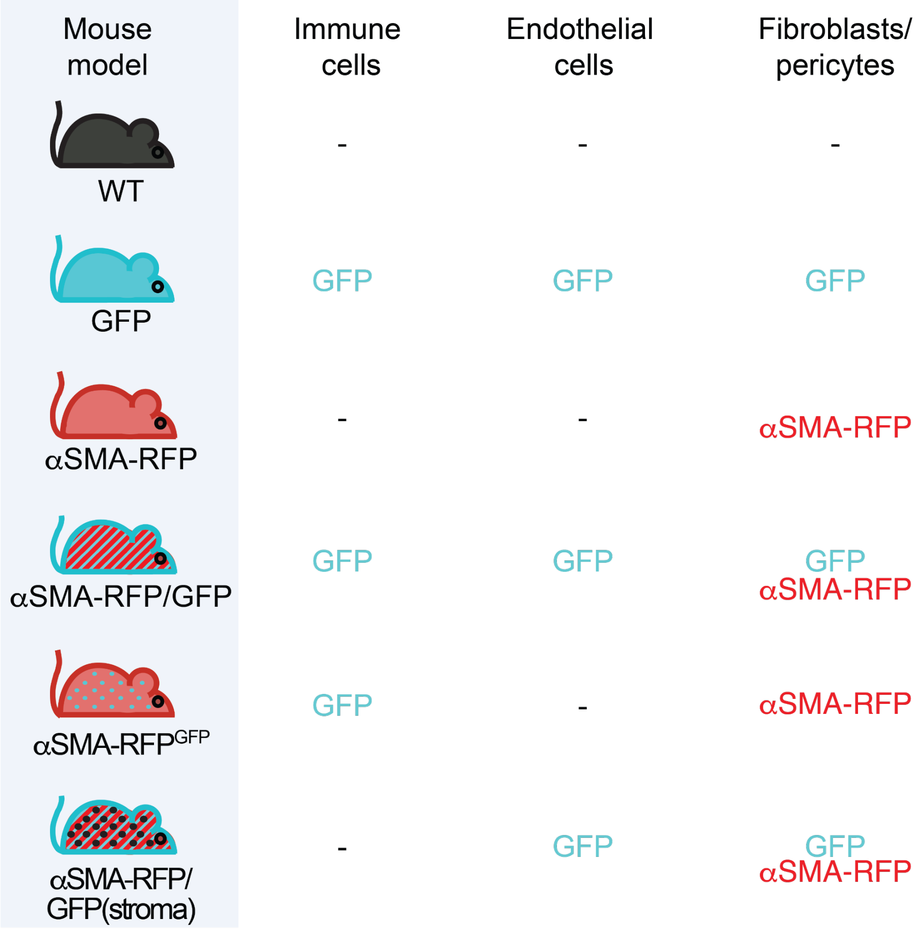

Fig. S1 Legend of the mouse models applied in the study.
Black mouse, C57BL/6 WT mouse, without any fluorescent cell; Cyan mouse, C57BL/6 UBC-GFP mouse, expressing GFP in each cell; Red mouse, C57BL/6 (Acta2-RFP)1Rkl/J mouse, expressing RFP in all the cells which produce αSMA (activated fibroblasts and pericytes); Cyan mouse with red stripes, αSMA-RFP/GFP mouse, expressing GFP in each cell and RFP in every cell expressing αSMA; Red mouse with cyan dots, αSMA-RFP^{GFP} mouse, showing GFP^{+} immune cells and RFP^{+} activated fibroblasts; Cyan mouse with red stripes and black dots, αSMA-RFP/GFP(stroma) mouse, showing non-immune GFP^{+} and RFP^{+} activated αSMA^{+} stromal cells.

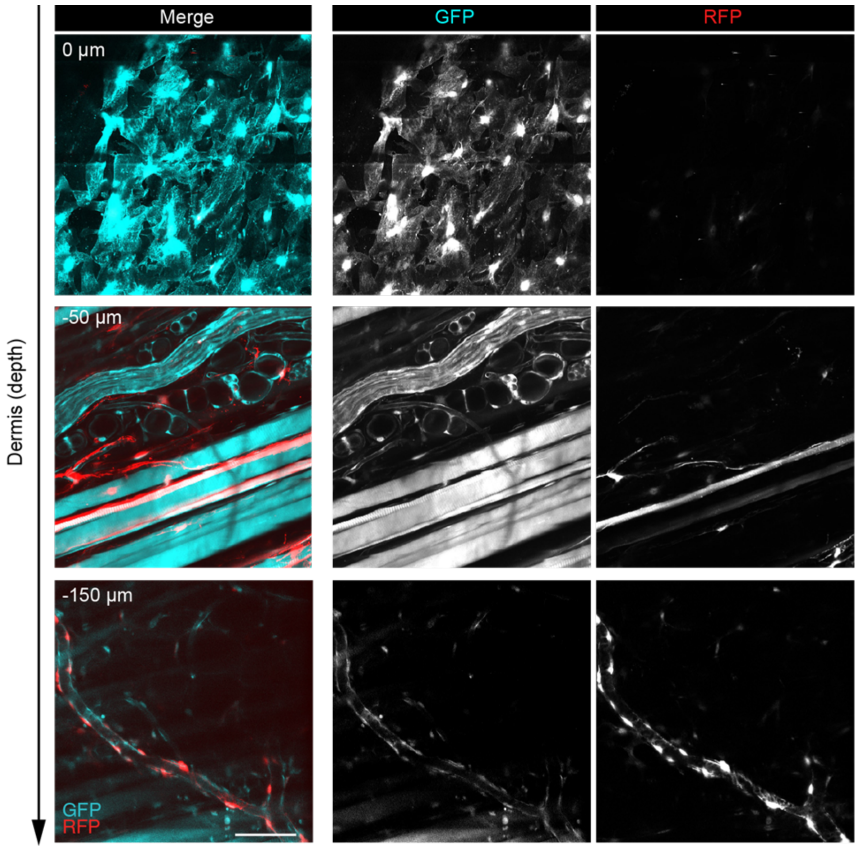

Fig. S2 Intravital imaging of implantation site in an αSMA-RFP/GFP(stroma) mouse.
Merged multiparameter and single-channel representations of GFP^+ cells (cyan) and RFP^+ cells (red) of three different layers of the dermis at the implantation site. Upper fascia (0µm of depth) showing GFP^+ inactive fibroblasts; Panniculus carnosus (-50µm of depth) showing GFP^+ nerves, adipocytes, and muscle fibers, together with RFP^+ muscle fibers, and activated fibroblasts; Subcutis (-150µm of depth) showing RFP^+ pericytes and GFP^+ endothelial cells around vessels. Scale bar, 100 µm.

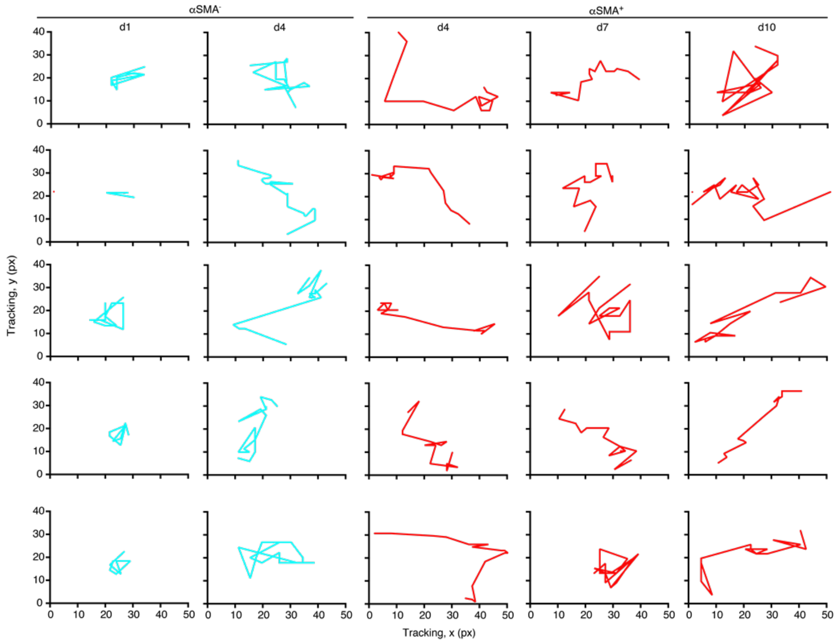

Fig. S3 Cell tracks.
XY plots representing examples of tracks of non-activated and activated fibroblasts monitored by time-lapse multiphoton microscopy at different time points (day 1, 4, 7, and 10).

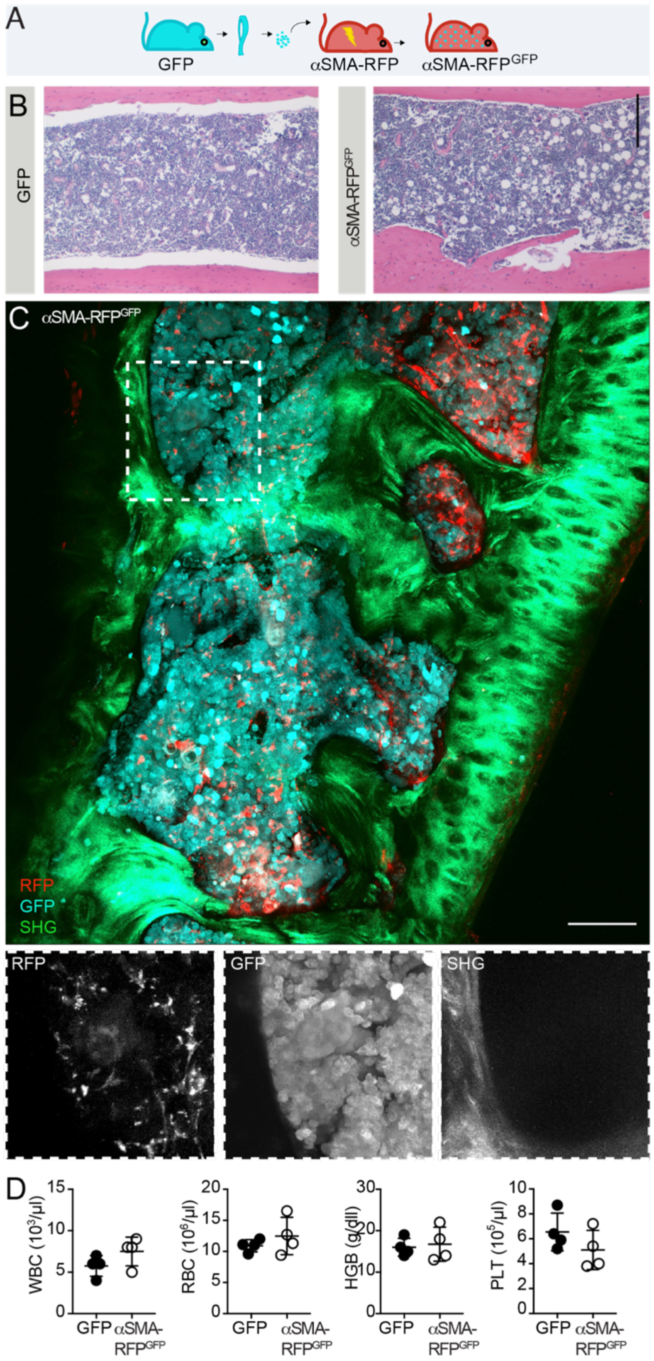

Fig. S4 Generation of a dual-color αSMA-RFP^{GFP} mouse through bone marrow transplant.
A, Schematic representation of the bone marrow transplant procedure resulting in a mouse with GFP^{+} bone marrow-derived immune cells and RFP^{+} myofibroblasts (αSMA-RFP^{GFP}).
B, H&E staining of a C57BL/6 GFP mouse bone marrow (left) and of an αSMA-RFP^{GFP} transplanted mouse (right). Scale bar, 100 µm.
C, Immunofluorescence analysis of a bone from a αSMA-RFP^{GFP} mouse. Overview with merged channels. Dashed box, inset; insets show single channels. Scale bar, 50 µm.
D, Circulating white blood cells (WBC), red blood cells (RBC), hemoglobin (HGB), hematocrit (HCT) and platelets (PLT) as monitored 30 days post-bone marrow transplant. Mean $\pm$ SD. No significant differences were identified by unpaired two-tailed Student's t-test.

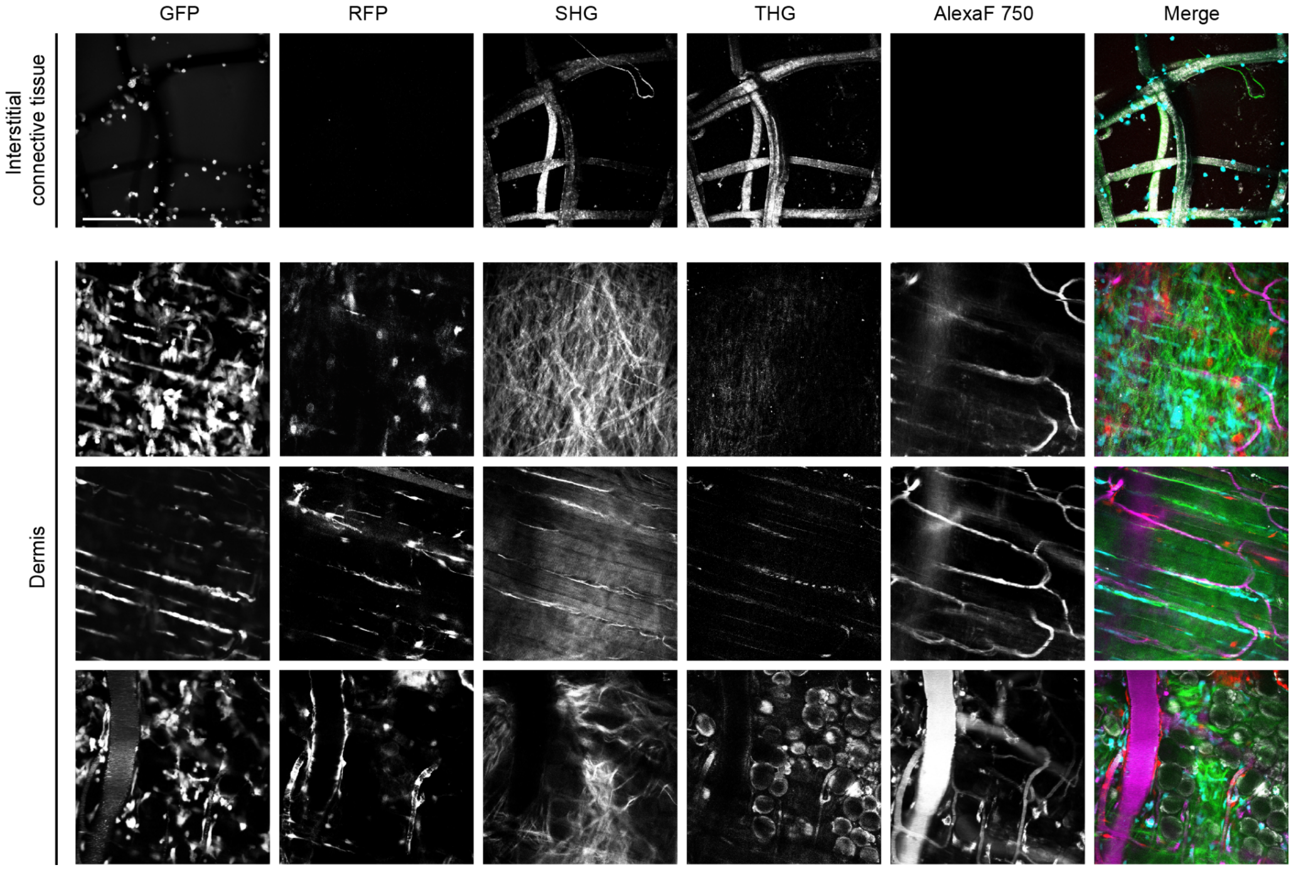

Fig. S5 Intravital imaging of PCL implant site in αSMA-RFP/GFP mouse.
A, B, 3D reconstruction of the implantation site up to 300 µm deep from the cover glass of the chamber was performed (the depth of imaging in relation to the position of the cover-glass is shown on the right). Merged and single-channel representations of the scaffold 1-day post-implantation (A) Upper fascia, panniculus carnosus and subcutis underlying the scaffold (B). GFP-positive cells (cyan); RFP-positive cells (red); collagen and scaffold, SHG (green); scaffold, THG (grey); vessels, Alexa Fluor 750 (magenta). Scale bar, 100 µm.

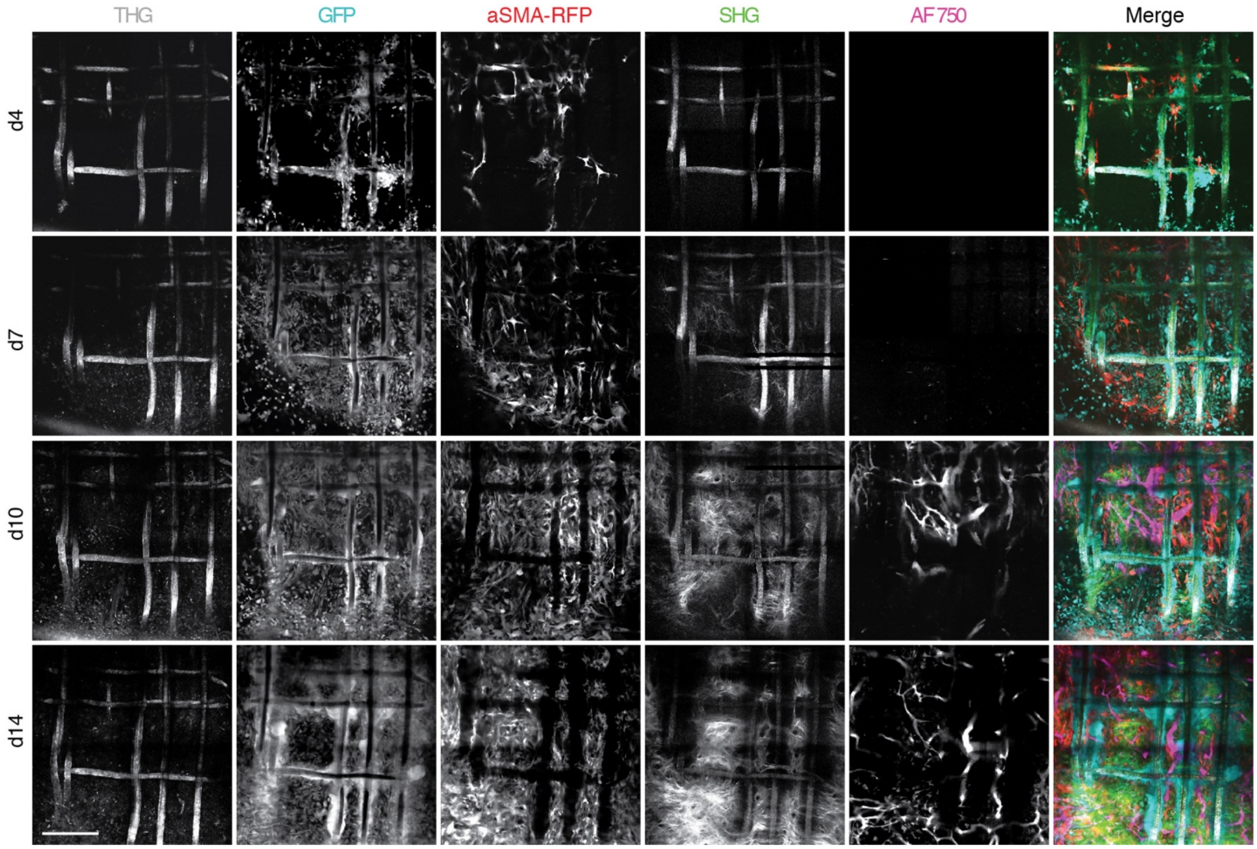

Fig. S6 Longitudinal intravital imaging of FBR at days 4, 7, 14 and 21 performed for the same lesion and subregion.

Extended version of Fig. 3A. Single-channel representations of scaffold fiber (THG), GFP-positive infiltrate cells, RFP-positive cells, SHG, detecting PCL fibers and fibrillar collagen and dextran-positive blood vessels. Merged multiparameter images on the right. Scale bar, 100µm.

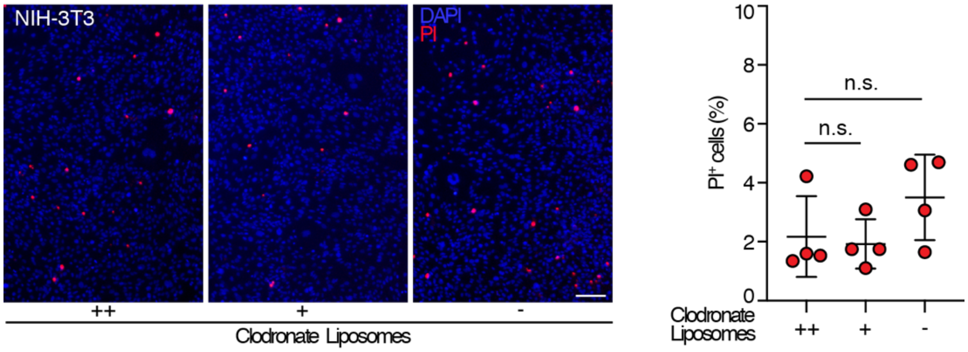

Fig. S7 Effect of clodronate liposomes on fibroblasts, *in vitro*.
Total nuclei (Hoechst) and dead cell (propidium iodide, PI) staining of NIH-3T3 cells treated with two different doses of clodronate liposomes (1:100, ++; 1:200, +) versus control-treated cells. Scale bar, 100µm; n.s., non-significant difference based on one-way analysis of variance followed by Tukey's HSD post hoc test.

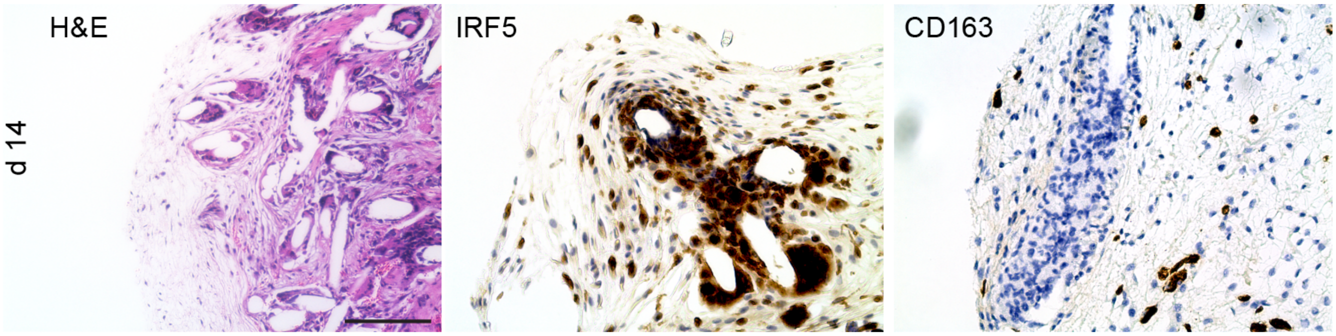

Fig. S8 Characterization of the PCL elicited FBR by histology.
Histology, (H&E staining) and IRF5 and CD163 expression detected by immunohistochemistry of the FBR in response to the PCL scaffold implantation 14 days post-implantation. Scale bar, 100µm.

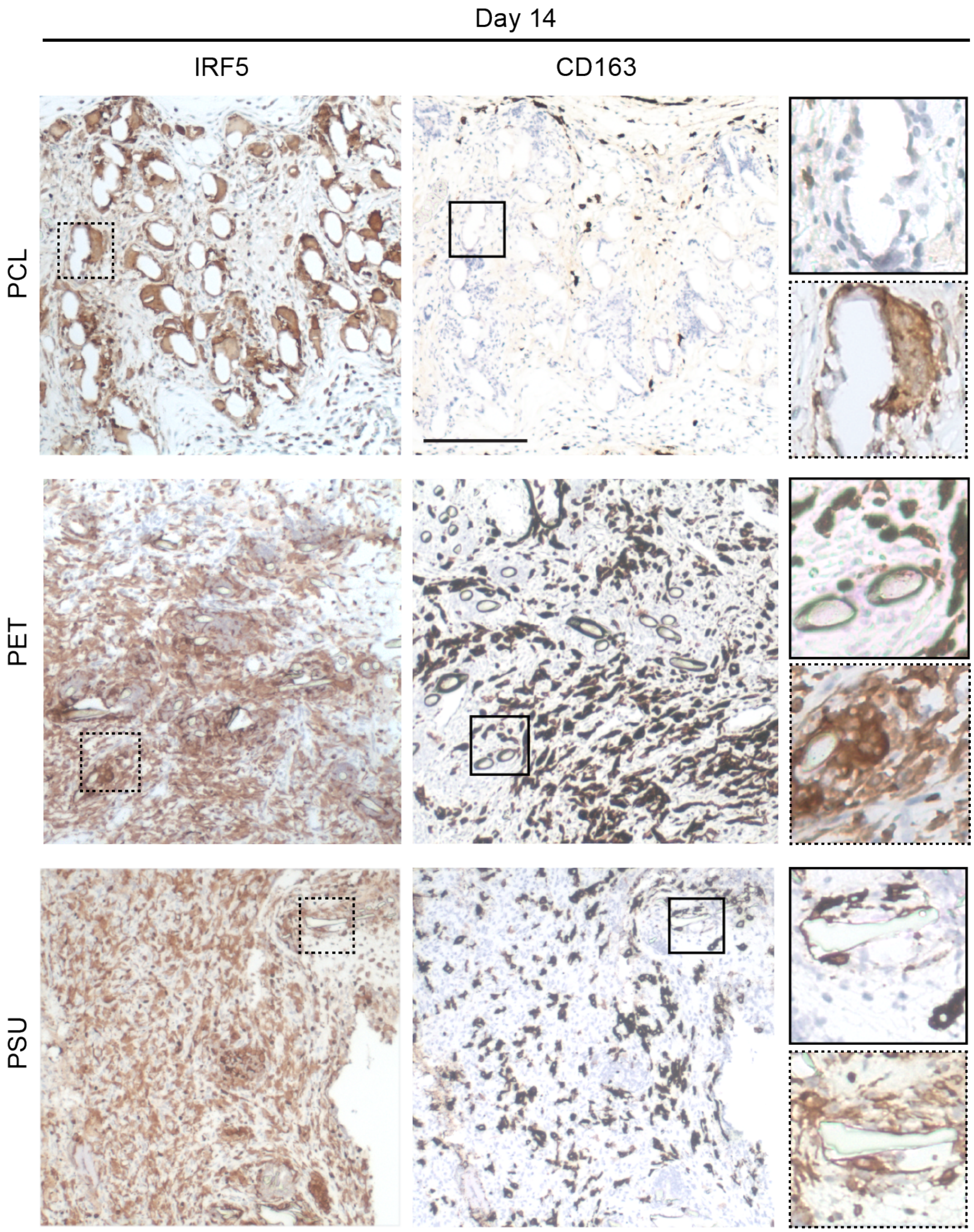

Fig. S9 M1 and M2 macrophages polarization around the three biomaterials fibers over time. IRF5 and CD163 expression detected by immunohistochemistry at day 14 post-implantation. Box, inset; inset, magnifications. n=3 scaffolds/group. Scale bar, 100 µm.

Supplementary movies

Supplementary Movie 1. Examination of αSMA negative and positive fibroblasts speed through iMPM. The region shows part of the edge of the scaffold and part of the implant-free dermis, 4 days after the surgery. Cyan, GFP^+ cells; red, RFP^+ cells. Time interval, 7 min; total time-lapse duration, 3 hours.

Supplementary Movie 2. Detail of an αSMA^+ fibroblast in a region close to the scaffold implantation site 4 days after surgery monitored by iMPM. Time interval between frames, 7 min; total time-lapse duration, 3 hours.

Supplementary Movie 3. Detail of an αSMA^- fibroblast in a region close to the scaffold implantation site 4 days after surgery monitored by iMPM. Time interval between frames, 7 min; total time-lapse duration, 3 hours.
